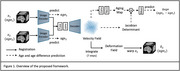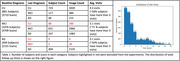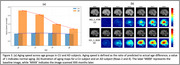# Revealing Brain Aging Trajectories Using Explainable Deep Learning on Longitudinal MRI Data

**DOI:** 10.1002/alz70861_108718

**Published:** 2025-12-23

**Authors:** Yuanwang Zhang, Hongming Li, Yong Fan

**Affiliations:** ^1^ University of Pennsylvania, Philadelphia, PA USA; ^2^ Perelman School of Medicine, University of Pennsylvania, Philadelphia, PA USA

## Abstract

**Background:**

Characterizing typical brain aging trajectories is crucial for detecting accelerated or resilient aging patterns. Existing deep learning methods lack mechanistic understanding of aging patterns. Although post‐hoc interpretation methods like saliency and class activation maps can be used to visualize aging patterns, they typically yield low‐resolution maps that obscure fine‐grained morphological changes and offer limited insight of the prediction process. To overcome these limitations, we develop an explainable deep learning framework to characterize local and global aging patterns jointly with direct interpretability on the underlying brain morphological changes.

**Method:**

Our framework learns morphological changes predictive of aging by jointly characterizing voxel‐wise brain changes and brain‐wise aging patterns. The voxel‐wise longitudinal brain changes are quantified with Jacobian determinant of deformation fields that are computed using a diffeomorphic registration method. To characterize whole‐brain morphological changes with aging, we introduce an aging map that acts as a spatial weighting function of the Jacobian determinants for predicting age difference between two scans, as illustrated in Figure 1. Specifically, the aging map reflects the relevance of each voxel to age‐related morphological changes. The prediction of age difference is further enhanced by the prediction of brain age of each individual scan as an auxiliary task. The method has been validated on the longitudinal ADNI dataset, comprising 10,579 scans from 2,366 subjects. All MRI scans undergo standard preprocessing, including bias field correction, skull stripping, and affine registration to MNI space. The model was trained on cognitively unimpaired (CU) individuals for learning typical aging trajectories.

**Result:**

Figure 3 (a) shows brain aging speed, the ratio of predicted to chronological age differences, of CU individuals and Alzheimer’s disease (AD) patients, with AD patients exhibiting substantially accelerated aging across all age groups. Figure 3 (b) illustrates the learned aging maps of a CU individual and an AD patient, highlighting that the AD patent had a more widespread distribution of age‐related morphological changes.

**Conclusion:**

Our method accurately predicts age differences and captures meaningful spatially distributed aging patterns, showing sensitivity to abnormal aging in AD patients. It offers an interpretable and effective framework for modeling brain aging trajectories.